# Supplementary material for: Role of a ZF-HD Transcription Factor in miR157-Mediated Feed-Forward Regulatory Module That Determines Plant Architecture in Arabidopsis
Source: Int J Mol Sci. 2022 Aug 4;23(15):8665. doi: 10.3390/ijms23158665 (PMC9369202; doi:10.3390/ijms23158665)
Supplement: Supplementary file 1 [file ijms-23-08665-s001.zip › Suppl Figure_ijms(0).pdf]

Figure S1

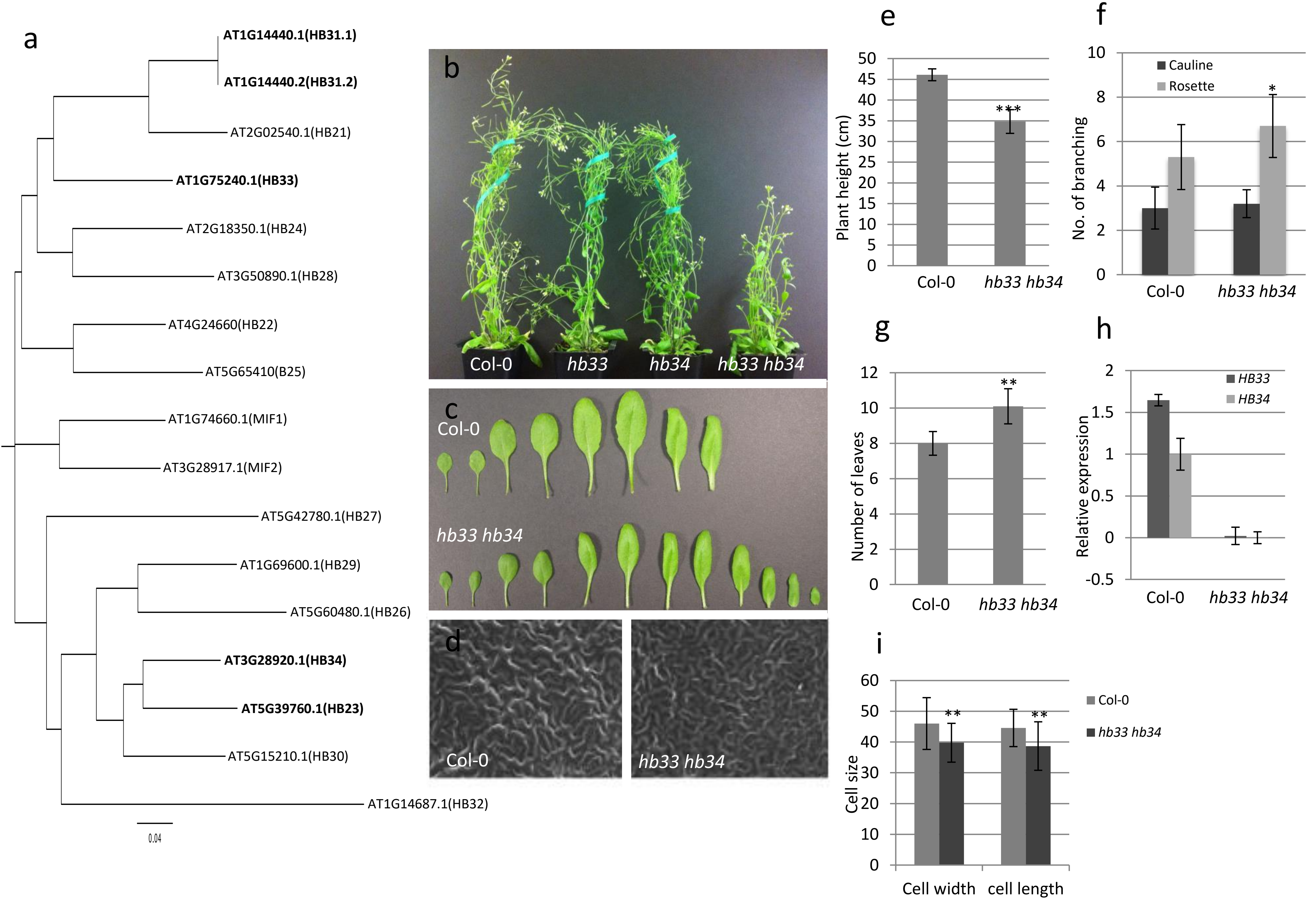

a
AT1G14440.1(HB31.1)
AT1G14440.2(HB31.2)
AT2G02540.1(HB21)
AT1G75240.1(HB33)
AT2G18350.1(HB24)
AT3G50890.1(HB28)
AT4G24660(HB22)
AT5G65410(B25)
AT1G74660.1(MIF1)
AT3G28917.1(MIF2)
AT5G42780.1(HB27)
AT1G69600.1(HB29)
AT5G60480.1(HB26)
AT3G28920.1(HB34)
AT5G39760.1(HB23)
AT5G15210.1(HB30)
AT1G14687.1(HB32)
0.04
b
Col-0
hb33
hb34
hb33 hb34
c
Col-0
hb33 hb34
d
Col-0
hb33 hb34
e
Plant height (cm)
50
45
40
35
30
25
20
15
10
5
0
***
Col-0
hb33 hb34
f
No. of branching
10
8
6
4
2
0
Cauline
Rosette
*
Col-0
hb33 hb34
g
Number of leaves
12
10
8
6
4
2
0
**
Col-0
hb33 hb34
h
Relative expression
2
1.5
1
0.5
0
-0.5
HB33
HB34
Col-0
hb33 hb34
i
Cell size
60
50
40
30
20
10
0
**
**
Col-0
hb33 hb34
Cell width
cell length

Figure S2

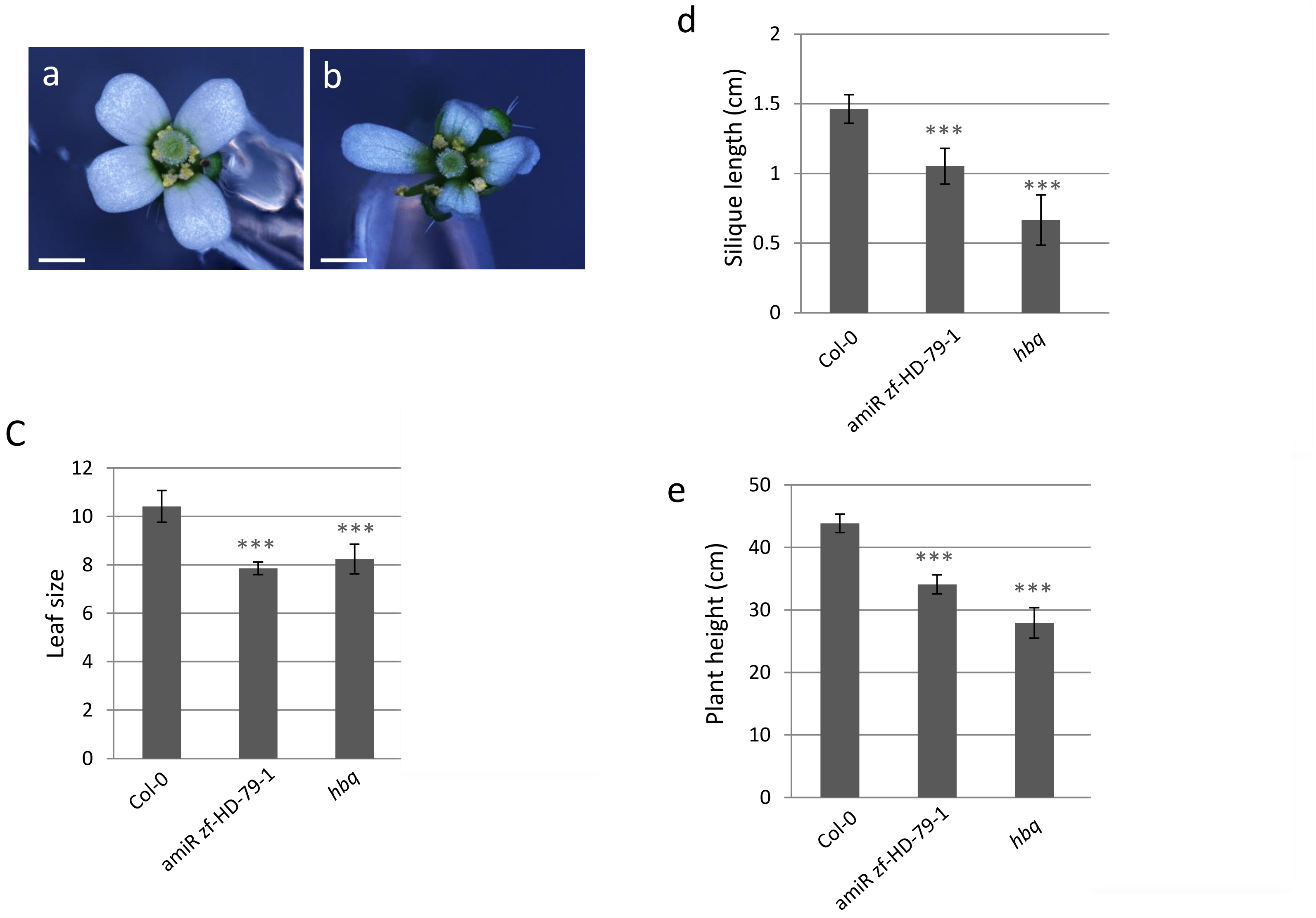

a
b
d
2
1.5
1
0.5
0
Silique length (cm)
***
***
Col-0
amiR zf-HD-79-1
hbq
C
12
10
8
6
4
2
0
Leaf size
***
***
Col-0
amiR zf-HD-79-1
hbq
e
50
40
30
20
10
0
Plant height (cm)
***
***
Col-0
amiR zf-HD-79-1
hbq

Figure S3

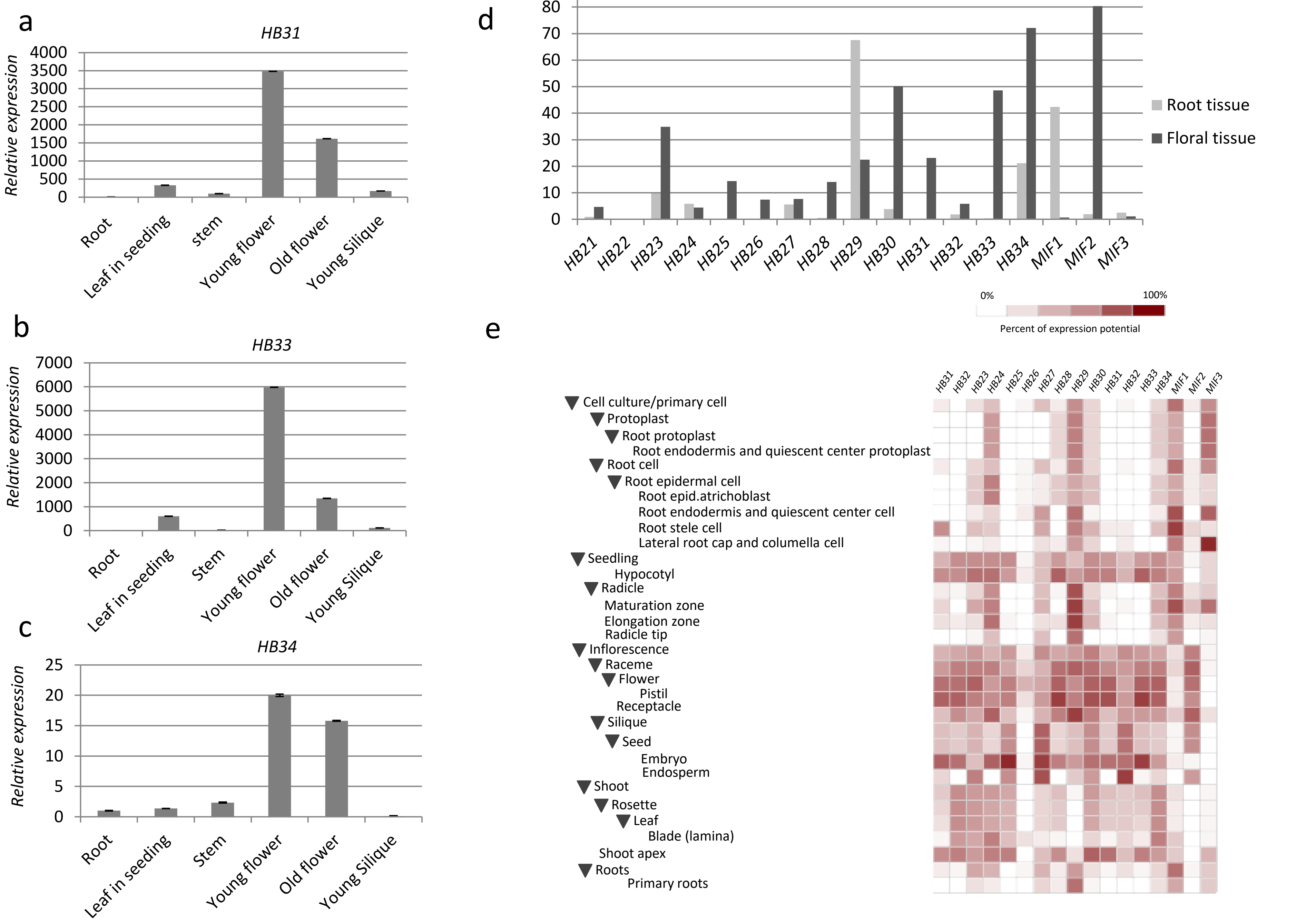
a
HB31
Relative expression
4000
3500
3000
2500
2000
1500
1000
500
0
Root
Leaf in seeding
stem
Young flower
Old flower
Young Silique
b
HB33
Relative expression
7000
6000
5000
4000
3000
2000
1000
0
Root
Leaf in seeding
Stem
Young flower
Old flower
Young Silique
c
HB34
Relative expression
25
20
15
10
5
0
Root
Leaf in seeding
Stem
Young flower
Old flower
Young Silique
d
90
80
70
60
50
40
30
20
10
0
HB21
HB22
HB23
HB24
HB25
HB26
HB27
HB28
HB29
HB30
HB31
HB32
HB33
HB34
MIF1
MIF2
MIF3
Root tissue
Floral tissue
e
0%
100%
Percent of expression potential
HB31 HB32 HB23 HB24 HB25 HB26 HB27 HB28 HB29 HB30 HB31 HB32 HB33 HB34 MIF1 MIF2 MIF3
Cell culture/primary cell
Protoplast
Root protoplast
Root endodermis and quiescent center protoplast
Root cell
Root epidermal cell
Root epid.atrichoblast
Root endodermis and quiescent center cell
Root stele cell
Lateral root cap and columella cell
Seedling
Hypocotyl
Radicle
Maturation zone
Elongation zone
Radicle tip
Inflorescence
Raceme
Flower
Pistil
Receptacle
Silique
Seed
Embryo
Endosperm
Shoot
Rosette
Leaf
Blade (lamina)
Shoot apex
Roots
Primary roots

Figure S4

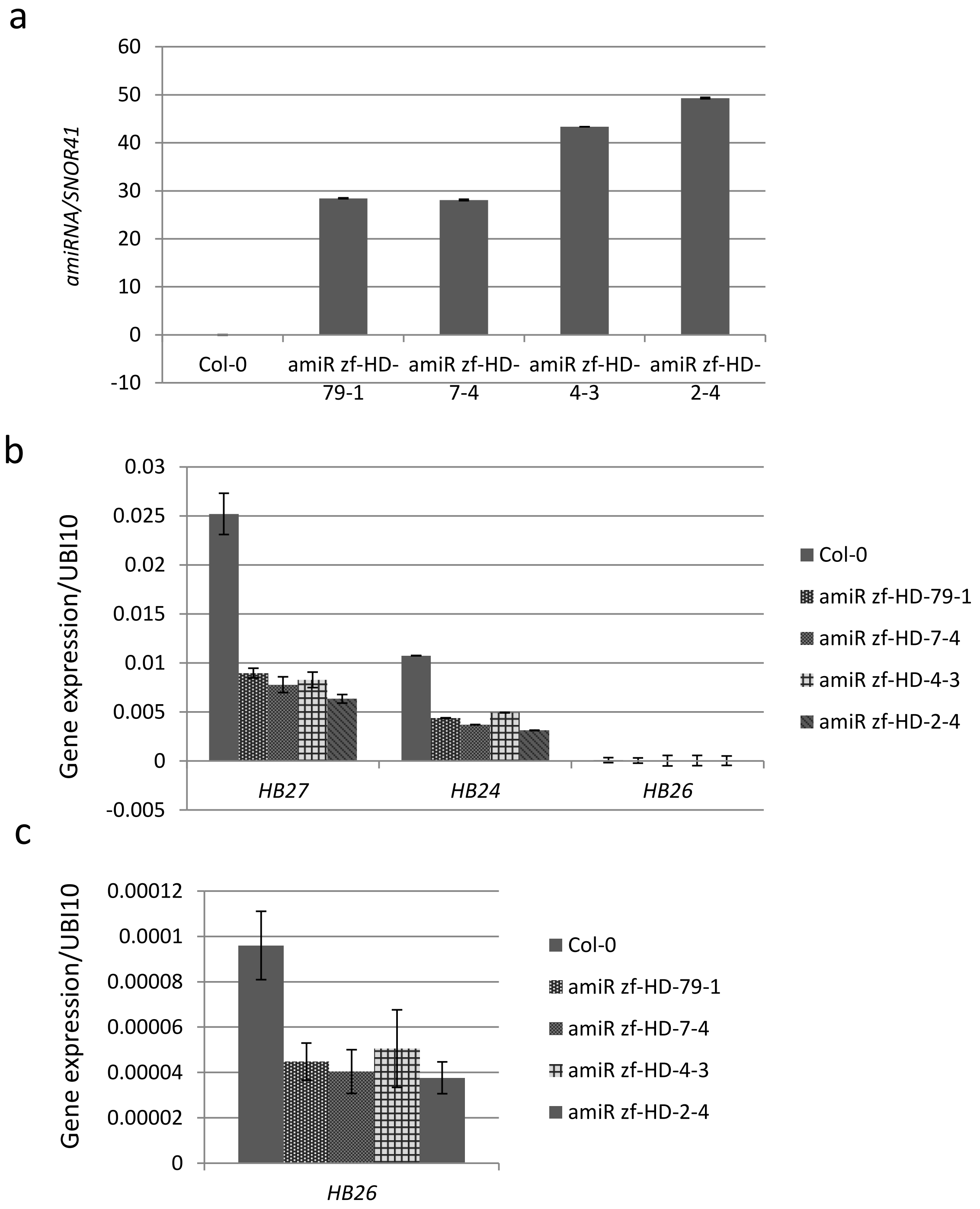
a
60
50
40
30
20
10
0
-10
amiRNA/SNOR41
Col-0
amiR zf-HD-79-1
amiR zf-HD-7-4
amiR zf-HD-4-3
amiR zf-HD-2-4
b
0.03
0.025
0.02
0.015
0.01
0.005
0
-0.005
Gene expression/UBI10
HB27
HB24
HB26
Col-0
amiR zf-HD-79-1
amiR zf-HD-7-4
amiR zf-HD-4-3
amiR zf-HD-2-4
c
0.00012
0.0001
0.00008
0.00006
0.00004
0.00002
0
Gene expression/UBI10
HB26
Col-0
amiR zf-HD-79-1
amiR zf-HD-7-4
amiR zf-HD-4-3
amiR zf-HD-2-4

Figure S5

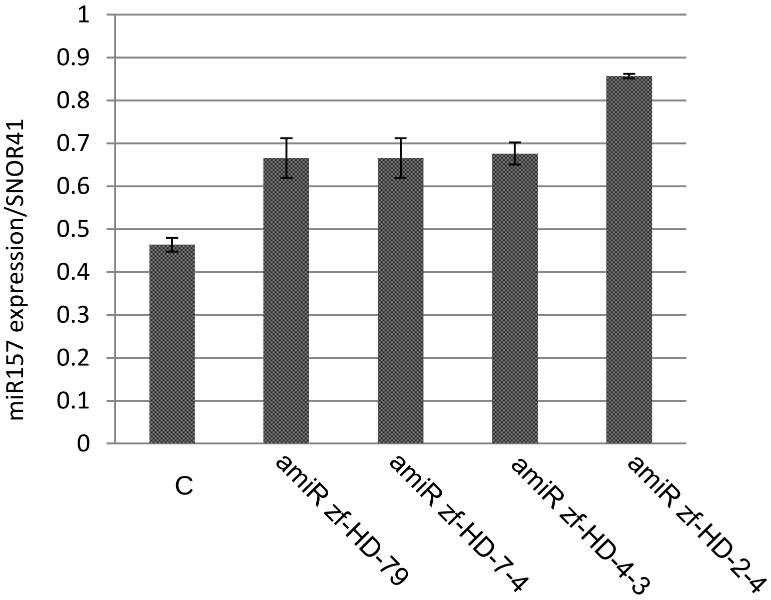
miR157 expression/SNOR41
1
0.9
0.8
0.7
0.6
0.5
0.4
0.3
0.2
0.1
0
C
amiR zf-HD-79
amiR zf-HD-7-4
amiR zf-HD-4-3
amiR zf-HD-2-4

Figure S6

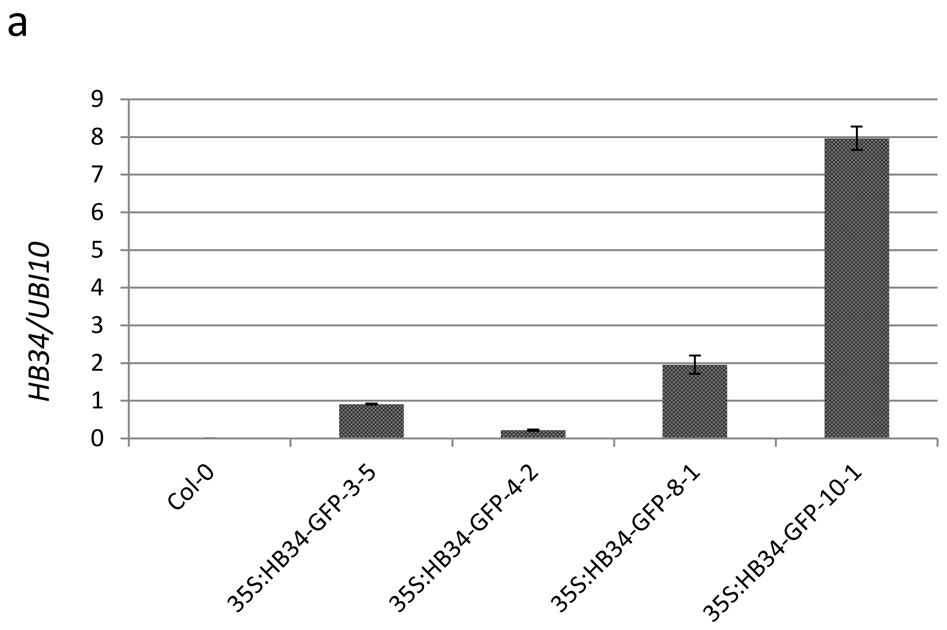
a
9
8
7
6
5
4
3
2
1
0
HB34/UBI10
Col-0
35S:HB34-GFP-3-5
35S:HB34-GFP-4-2
35S:HB34-GFP-8-1
35S:HB34-GFP-10-1

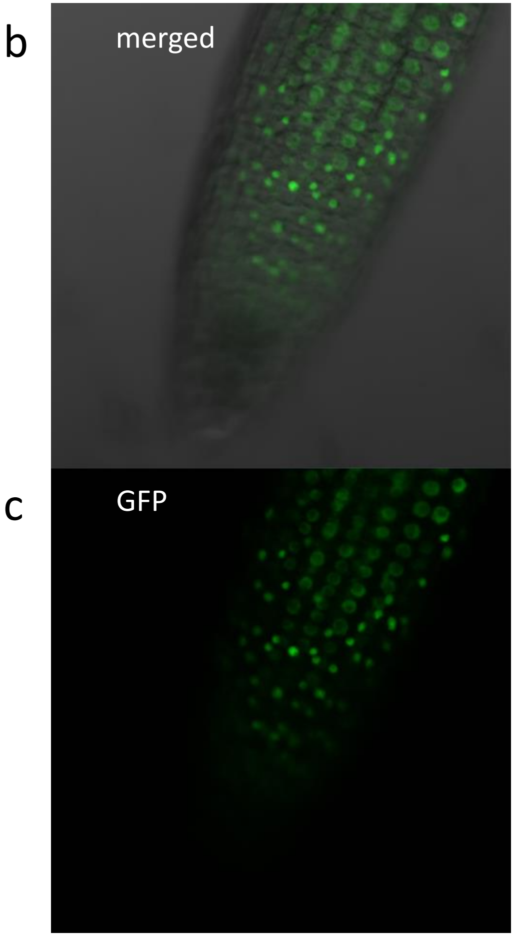
b
merged
c
GFP

Figure S7

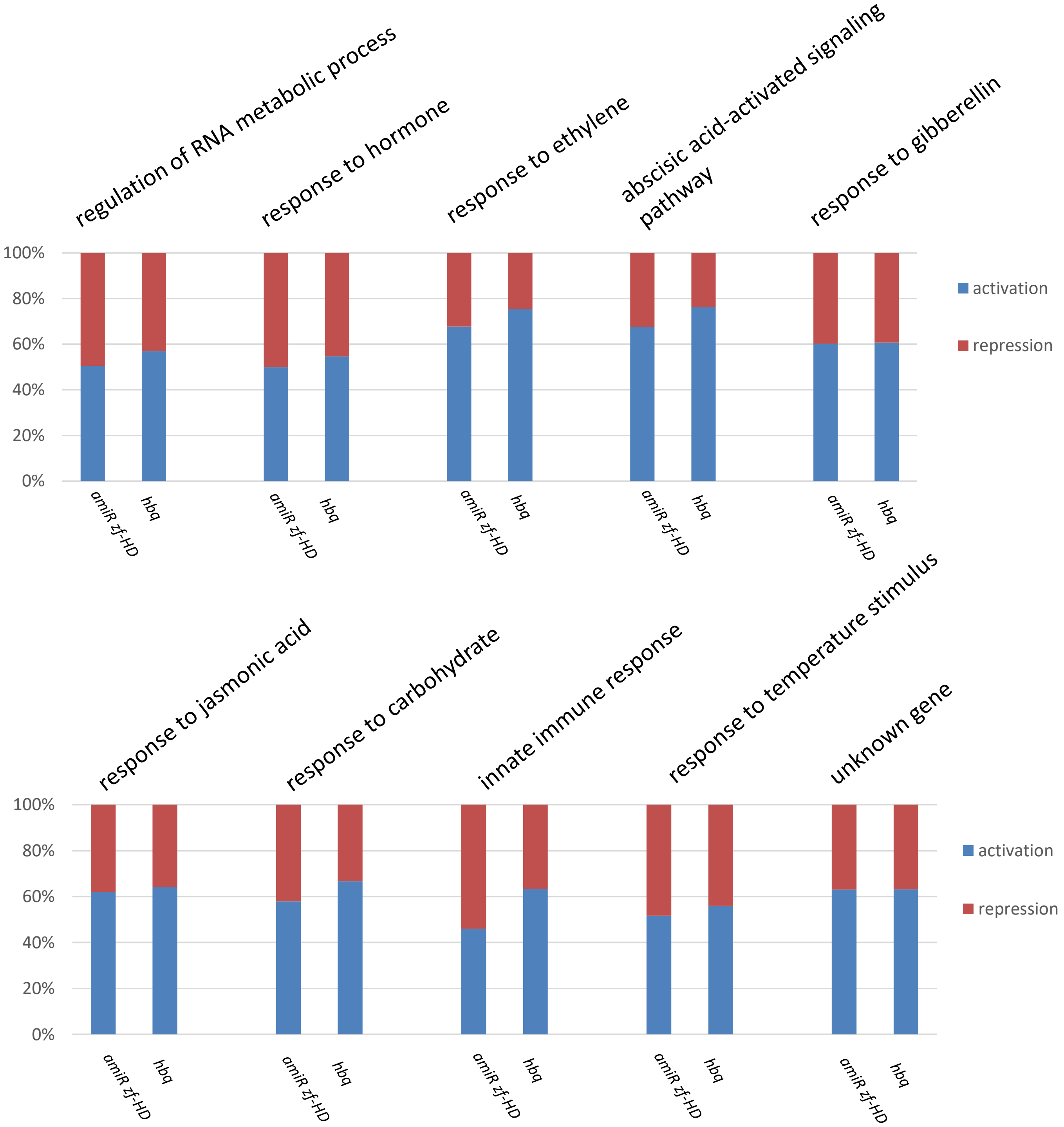
regulation of RNA metabolic process
response to hormone
response to ethylene
abscisic acid-activated signaling pathway
response to gibberellin
100%
80%
60%
40%
20%
0%
amiR zf-HD
hbq
activation
repression
response to jasmonic acid
response to carbohydrate
innate immune response
response to temperature stimulus
unknown gene

Figure S8

a

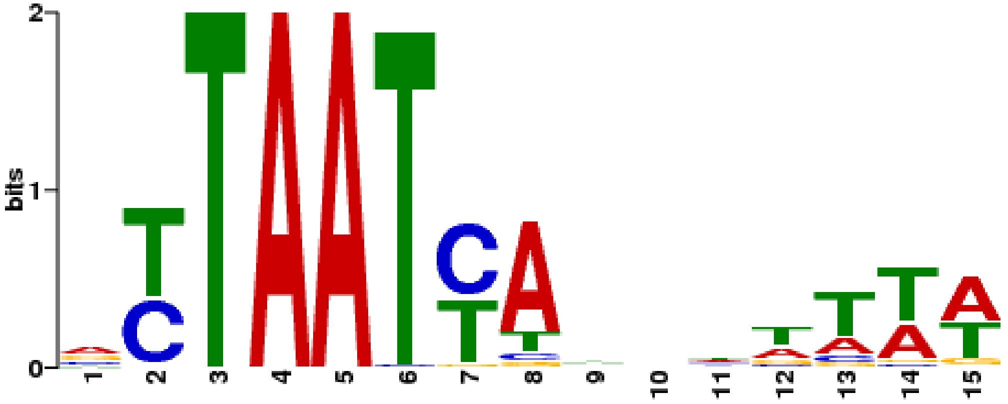

2
1
0
bits
1
2
3
4
5
6
7
8
9
10
11
12
13
14
15

b

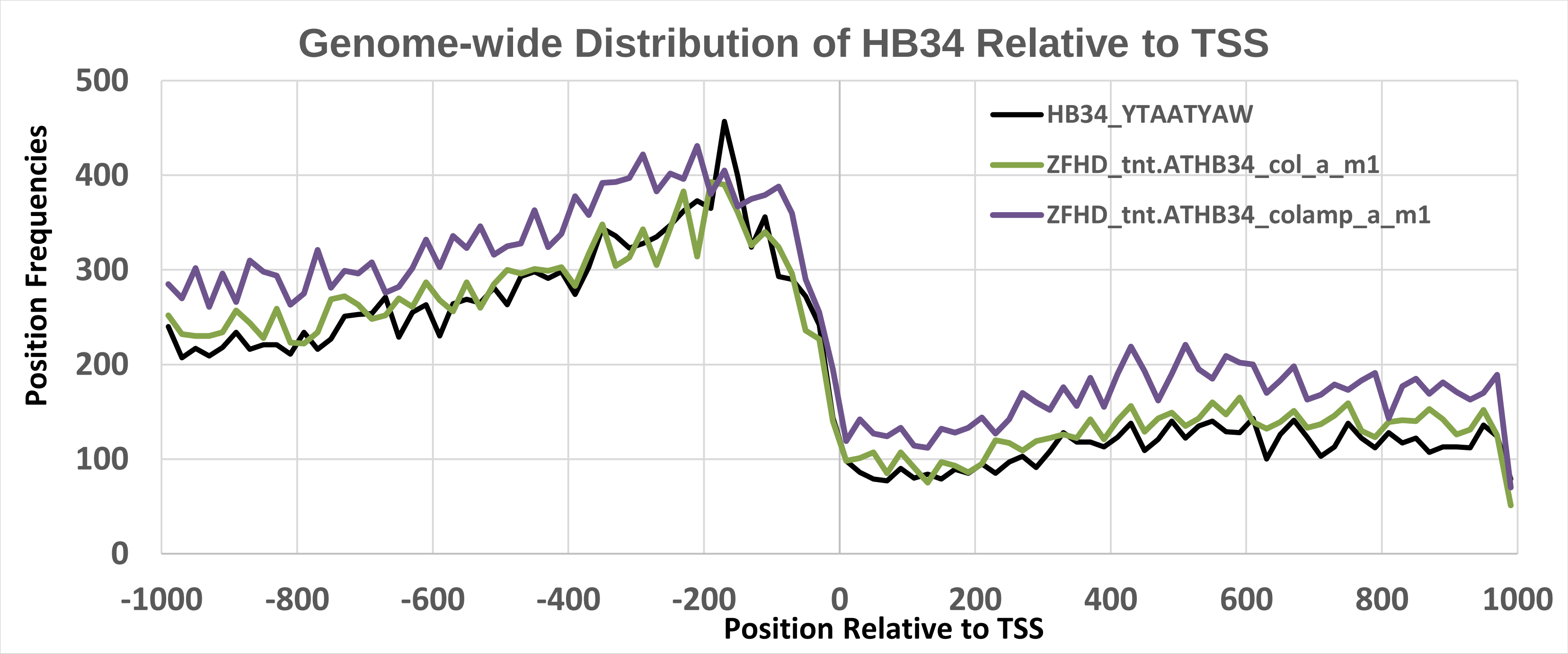

Genome-wide Distribution of HB34 Relative to TSS
500
400
300
200
100
0
Position Frequencies
-1000
-800
-600
-400
-200
0
200
400
600
800
1000
Position Relative to TSS
HB34_YTAATYAW
ZFHD_tnt.ATHB34_col_a_m1
ZFHD_tnt.ATHB34_colamp_a_m1

Figure S9

a

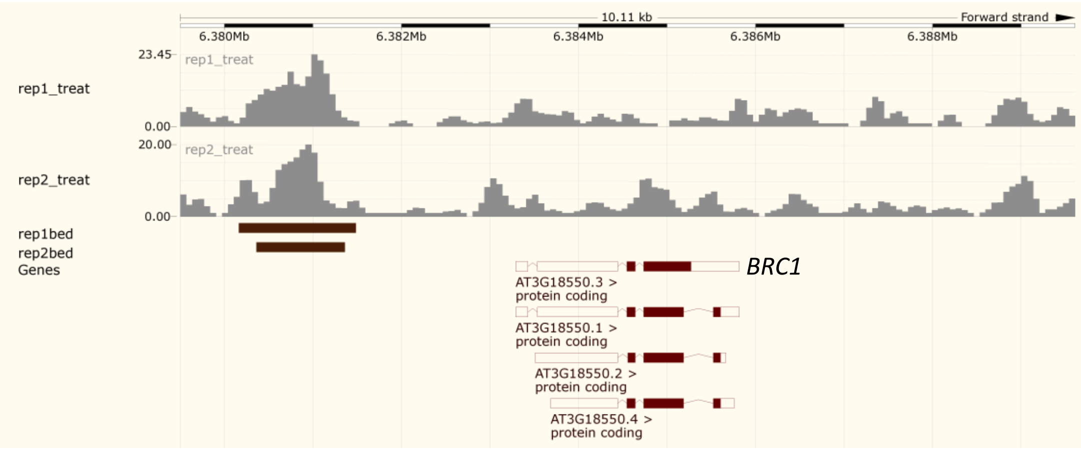

b

TF family members	Gene id	amiR zf-HD	*hbq*	Gene name
TCP	AT3G18550			BRC1
HD-ZIP	AT1G17920			HDG12
HD-ZIP	AT1G26960			AtHB23
HD-ZIP	AT1G27045			ATHB-54
HD-ZIP	AT1G30490			PHV
HD-ZIP	AT1G52150			ATHB-15
HD-ZIP	AT1G69780			ATHB13
HD-ZIP	AT1G70920			ATHB18
HD-ZIP	AT1G73360			HDG11
HD-ZIP	AT2G01430			ATHB17
HD-ZIP	**AT2G18550**			**HB-2(HB21)**
HD-ZIP	AT2G22430			ATHB6
HD-ZIP	AT2G22800			HAT9
HD-ZIP	AT2G32370			HDG3
HD-ZIP	AT2G44910			ATHB4
HD-ZIP	AT2G46680			ATHB-7
HD-ZIP	AT3G01220			ATHB20
HD-ZIP	AT3G01470			ATHB-1
HD-ZIP	AT3G60390			HAT3
HD-ZIP	AT3G61890			ATHB-12
HD-ZIP	AT4G00730			ANL2
HD-ZIP	AT4G04890			PDF2
HD-ZIP	AT4G16780			ATHB-2
HD-ZIP	AT4G17460			HAT1
HD-ZIP	AT4G21750			ATML1
HD-ZIP	AT4G25530			FWA
HD-ZIP	AT4G32880			ATHB-8
HD-ZIP	**AT4G36740**			**HB-5(HB40)**
HD-ZIP	AT4G37790			HAT22
HD-ZIP	AT4G40060			ATHB16
HD-ZIP	AT5G06710			HAT14
HD-ZIP	AT5G15150			ATHB-3
HD-ZIP	AT5G46880			HB-7
HD-ZIP	AT5G47370			HAT2
HD-ZIP	AT5G60690			REV
HD-ZIP	AT5G65310			ATHB5
HD-ZIP	**AT5G66700**			**HB53**

c

TF family members	Gene id	amiR zf-HD	*hbq*	Gene name
bZIP	AT4G34000			ABF3
bZIP	AT2G46270			GBF3
bZIP	AT2G36270			ABI5
NAC	AT1G69490			NAP
