# Supplementary material for: Role of a ZF-HD Transcription Factor in miR157-Mediated Feed-Forward Regulatory Module That Determines Plant Architecture in Arabidopsis
Source: Int J Mol Sci. 2022 Aug 4;23(15):8665. doi: 10.3390/ijms23158665 (PMC9369202; doi:10.3390/ijms23158665)
Supplement: Supplementary file 1 [file ijms-23-08665-s001.zip › Supplementary Materials Captions.pdf]

Figure S1. Phenotypes of *hb33 hb34* double loss-of-function mutants. (a) Phylogenetic analysis of the ZF-HD gene family in *Arabidopsis*. The phylogenetic tree was generated by gramene [69]. (b) Plant morphology in Col-0, *hb33*, *hb34*, and *hb33 hb34*. (c) Leaf morphology in Col-0 and *hb33/hb34*. (d) SEM picture showing leaf cell morphology in Col-0 and *hb33 hb34*. (e) Plant height of Col-0 and *hb33 hb34*. (f) Number of branches of Col-0 and *hb33 hb34*. (g) Number of leaves of Col-0 and *hb33/hb34*. (h) Expression of *HB33* and *HB34* was monitored by qRT-PCR in Col-0 and *hb33/hb34*. (i) Epidermal cell size in the leaf length and leaf width direction in Col-0 and *hb33/hb34*.

Figure S2. Phenotypic comparison between Col-0 and *zf-hd* mutants. (a-b) Floral morphology in Col-0 (a) *hbq* (b). (c) Leaf size in Col-0, amiR zf-HD 79-1, and *hbq*. (d) Silique length n Col-0, amiR zf-HD 79-1, and *hbq*. (e) Plant height in Col-0, amiR zf-HD 79-1, and *hbq*.

Figure S3. Expression analysis of ZF-HD TF genes in various tissues including root, leaf in seedling, stem, young flower, old flower, and young silique. (a-c) qRT-PCR analysis to confirm expression of *HB31*, *HB33*, and *HB34*. Three biological replicates were used for qRT-PCR. (d) RNA-seq data for ZF-HD TF family members in young flower and root tissue. Two biological replicates were used for RNA-seq. (e) Publicly available microarray database of ZF-HD TF from GENEVESTIGATOR (https://genevestigator.com/, accessed on 1 June 2020).

Figure S4. Expression of amiRNA and targets in amiR zf-HD mutants.

Figure S5. Expression changes based on the up-regulated DE genes in *zf-hd* mutants.

Figure S6. Expression of *HB34* in 35S:*HB34-GFP*/*hb34* transgenic lines and HB34 localization in root tissue in the transgenic 35S:*HB34-GFP*/*hb34* transgenic line.

Figure S7. Relative proportion of activated/repressed genes associated with enriched GO terms among the directly bound targets of HB34.

Figure S8. Genome wide distribution of HB34 motifs. (a) Sequence logo of novel HB34 motif (b) position frequency distribution graph based on novel HB34 motif and two other PWMs for HB34 (ZFHD_tnt.ATHB34_col_a_m1; ZFHD_tnt.ATHB34_colamp_a_m1) obtained from the publicly available Plant Cistrome Database.

Figure S9. Genome-wide binding target analysis of HB34 reveals that HB34 directly regulates *BRC1*. (a) Genome browser view showing HB34 ChIP-seq reads aligned near *BRC1.* (b) Heat map of TCP and HD-ZIP TF in amiR zf-HD and *hbq* in comparison with WT. (c) Heat map of bZIP and NAC TF in amiR zf-HD and *hbq* in comparison with WT.

Table S1. Sequences of primers used for mutant genotyping, expression analysis of qRT-PCR, and transient assay vector construction.

Table S2. Expression changes of individual down- or up-regulated TF DEGs.

Table S3. Expression changes of miRNAs and miRNA targets in Col-0, amiR zf-HD, and *hbq*.

Table S4. Network motif analysis based on the RNA-seq. data and miRNA target.

Table S5. Molecular perturbation grouped by TF family that is directly bound and modulated by ZF-HD TFs in Col-0, amiR zf-HD, and *hbq*.

Table S6. Direct target genes of HB34 identified by ChIP-seq analysis.
